# 

**Published:** 1891-11-14

**Authors:** 


					MR INFANTS
AND INVALIDS
: W f&,
homas s Hospital
Glasgow Western Infirmary
ahcTNORWICH hospi tal
WITH EXTRA NURSING SUPPLEMENT.
w
No. 268. Tol. XI.] Saturday,
Novf.mbeb 14th, 1891.
[One Penny,

				

